# Guy's Hospital

**Published:** 1831-05

**Authors:** 


### HOSPITAL REPORTS.

### GUY'S HOSPITAL.

### *Removal of a Tumor, Fifty-six Pounds in Weight, extending from beneath the Umbilicus to the anterior Border of the Anus.**

Hoo Loo, a Chinese labourer, was admitted into Luke's ward, Guy's Hospital, in the third week of March last with an extraordinary tumor depending from the lower part of the abdomen, and of a nature and extent hitherto unseen in this country. He had been brought to England from Canton, by his own desire, in an East Indiaman, for the purpose of having this tumor removed at one of the London hospitals, the native surgeons declining to make the attempt, a general disinclination to the performance of serious operations prevailing in the East, where both the climate and the law offer important objections to surgical proceedings which may lead to the loss of human life. The case excited considerable interest, both in and out of the profession, from the first moment of his arrival, and he was visited in the hospital by a great number of persons of all ranks.

We have heard that, on his voyage here, the change of air had such an effect on his constitution, as to occasion a material increase in the tumor. Since his arrival, his appetite, health, and spirits, were extremely good. While in the hospital, there appeared nothing to induce the surgeon to order him any medicine. His diet consisted principally of boiled rice, and no restraint was placed on his appetite, which was very great. He was generally considered to have improved in health while in the hospital, though it was difficult to form a decided estimate on this point. He all along contemplated the operation with satisfaction.

It was generally understood that the operation would be performed on Tuesday last, but so great a crowd of spectators was apprehended, that Saturday, which is a *dies non* in the hospital, was fixed on instead. Notwithstanding this precaution, however, an assemblage, unprecedented in numbers on such an occasion, presented themselves for admission at the operating theatre, which was instantly filled in every part, although none but pupils, and of those only such as could at the moment present their " hospital tickets," were admitted. Hundreds of gentlemen were consequently excluded, and it became obvious to the officers of the hospital that some other room must be selected. Accordingly, Sir Astley Cooper entered, and, addressing the pupils, said, that, in consequence of the crowd, the patient being in a state which would admit of the removal, the operation would take place in the great anatomical theatre. A tremendous rush to that theatre accordingly took

* Lancet.

place, where accommodation was afforded to 680 persons, and where preparations were immediately made for the patient. In about a quarter of an hour, Hoo Loo entered, accompanied by two nurses and a *posse comitatus,* consisting of various functionaries of the hospital, and in the course of a few minutes he was secured on the operating table. A short consultation now took place between Sir A. Cooper, Mr. Key, and Mr. Callaway, during which it was finally agreed that, if it were found possible, the genital organs should be preserved. The face of the patient was then covered, and Mr. Key, taking his station in front of the tumor, commenced the operation. His object, apparently, was to make three flaps, of such a form and extent, as would cover the penis and the testes when they should be relieved from the tumor. The first flap was made on the anterior part of the tumor; the others on either side. The former of these would have covered the penis, and the two others (which were made semilunar in figure with the convexities looking outwards) would have accomplished the purpose, when united, of enveloping the testes, and at the same time of forming the integument of the perineum. The first incision was commenced on the right side, just under the abdominal ring, and being carried obliquely inwards for about an inch, was continued so as to form the semilunar cut, from the lowermost point of which, the knife was again carried inwards, in order, apparently, to leave such a small projection of integument as, with a corresponding piece on the opposite side, might serve to go round the root of the penis. This portion having been reserved, the incision was continued onwards for about four inches in a straight line, and was then turned at a right angle, parallel with the operator, forming a line of about two and a half inches in length. A similar cut was then made on the left side, and connected with that on the right by the parallel or transverse cut. The flap thus formed was now dissected from the tumor, and laid back upon the abdomen. The operator then proceeded to lay bare the two cords and the penis, a step in the operation which was performed with very great neatness.

Sufficient time had now elapsed for the depressing effects of the operation to exhibit themselves, while the penis and testicles had yet to be dissected out. The determination to attempt this arose from its having been ascertained that the sexual inclinations of the man were unimpaired, seminal emissions being occasionally experienced. The delay, however, which so intricate a portion of the operation would have occasioned, now induced Sir Astley Cooper to propose that the genital organs should be sacrificed; and the suggestion was promptly acceded to. A temporary ligature was accordingly passed round each of the cords and the penis, and, these being divided, the remainder of the operation was pursued solely with a view to the removal of the whole mass. But a period of time elapsed before the conclusion of the operation, which must have far exceeded the anticipations even of the most fearful; and, by the time the tumor was entirely separated, and the exposed parts were closed over, an hour and forty-four minutes had passed. This tremendous protraction was chiefly occasioned by the intervals which were, from time to time, allowed the patient for recovery from the fits of exhaustion which supervened. Complete syncope occurred twice, and, during the whole of the latter steps of the operation, he was in a state of fainting. The quantity of blood lost was variously estimated by those who assisted, and, though certainly not large, it was the operator's own impression that the hemorrhage was the immediate cause of death. It would probably be correct to state the loss at twenty-five ounces, although as few as fourteen, and as many as thirty, were named. Of this quantity, not more, we should think, than a single ounce was arterial: all the ligatures were quickly applied, and with great dexterity. The number of large veins divided was immense, but only three small arteries, besides the two spermatic, were taken up.

Immediately after the removal of the tumor, another fit of syncope (if syncope could be said to be at all incomplete for the last half-hour,) came on, from which the poor fellow did not for a moment rally. No remedies that were directed to overcome this state of collapse had the slightest effect: warmth and friction of the extremities, warmth to the scrobiculus cordis, the injection of brandy and water into the stomach, and, ultimately, from the suspicion that the loss of blood had been too great, transfusion to the amount of six ounces, taken from the arm of a student, (one amongst several who offered to afford blood,) were amongst the means resorted to. The heart's action gradually and perceptibly sunk. The patient did breathe after the operation, but that is as much as can be said. Artificial respiration was subsequently, but vainly attempted.

The fortitude with which this great operation was approached, and throughout undergone, by Hoo Loo, was, if not unexampled, at all events never exceeded in the annals of surgery. A groan now and then escaped him, and now and then a slight exclamation, and we thought we could trace in his tones a plaintive acknowledgment of the hopelessness of his case. Expressions of regret, too, that he had not rather borne with his affliction than suffered the operation, seemed softly but rapidly to vibrate from his lips as he closed his eyes, firmly set his teeth, and resignedly strung every nerve in obedience to the determination with which he had first submitted to the knife.

His character was naturally exceedingly amiable. When occupied in thought, his features assumed an appearance of slight melancholy, but at other times a very cheerful and good-tempered expression of countenance prevailed. The appearance of the features after death was very characteristic of this. Whenever an exhibition of the tumor was desired, he was displeased and somewhat reluctant, seeming to imply by his language, that it was of "no use" to shew it. With the nurses he had become a great favorite, and his death elicited the utmost commiseration, perhaps a few tears, in the ward which he inhabited.

Hoo Loo was thirty-two years of age, and the tumor had been ten years arriving at its present growth. Its effect upon his frame, and the muscles of the abdomen was not particularly oppressive. It of course occasioned a very great strain upon the fore part of the body, and to preserve his balance he was compelled to throw the shoulders backwards, and assume the gait of an alderman whose belly preponderates and displaces his centre of gravity. The weight of the tumor was conjectured to be about seventy pounds, but, when placed in the scales after its removal, it weighed but fifty-six. His strength was not affected by it. He could take a stout lad in his arms, and toss him with ease.

The tumor commenced in the prepuce; the cause of it is involved in obscurity; nothing could be gathered from the patient to lead to the supposition that it was the effect of a blow, or to justify its being ascribed to any one particular exciting cause. Its form inclined to that of a flattened spheroid. When removed, and resting either upon its upper surfaces or its base, it sunk and spread until its mean depth was about six inches. The neck or peduncle by which it was attached was of a triangular form; the upper and anterior side of the neck was parallel with the os pubis, extending on each side of the external abdominal ring about two inches and a half, its other sides occupying the lateral boundaries of the perineum, and meeting in an acute angle immediately before the anus. Although the disease commenced in the prepuce, that portion of the tumor, or an elongation of it, was preserved in an isolated state from the rest of the growth, although, of course, totally disfigured, and presenting far greater appearance of disease than other parts of the integument. It now hung at the lowermost portion of the tumor, forming a second tumor, presenting an orifice for the discharge of the urine, varying in its transverse diameters from one to three inches. The passage to the urethra was, as far as the finger could reach, smooth and hardened: externally, it was a mass of tubercles, which spread on each side over the surrounding portion of the tumor, presenting, for a considerable distance, a hard and thickened cutis, studded with elevations. The sides and posterior part of the body of the tumor, as well as the whole of the peduncle, were of a perfectly healthy and natural appearance. The circumference of the mass was four feet, that of the neck two feet, each side of the neck, which was about equilateral, measuring eight inches. When the patient stood upright, the lower part of the tumor reached considerably below the knees.

It may be mentioned, that the urine also found a vent externally by an opening situated on the surface of the tumor, immediately at the base of the preputial tumor; which latter, as it had a strong tendency to overlap on the side of the opening, hid the false passage, if it may be so called, from sight.

*Post-mortem examination.* The examination of the body took place on Thursday morning.

From the tumor itself, a certain quantity of fluid (not large) escaped during the operation: the solid mass weighed fifty-six pounds, as we have already mentioned: it was decidedly steatomatous; but the examination has not yet been completed. About an inch of the penis was laid bare by the dissection.

There were no appearances which arrested the attention on the examination of the body. The stomach, and other viscera, were perfectly healthy. The cremaster muscles were both much increased, apparently from an enlargement of the fibres, and not from any multiplication of them. The state of the wound was not at all unnatural; but some of the veins which came from the tumor were as large as the little finger. The arteries of the chord were not larger than usual.

